# Cationic Covalent Organic Polymer Thin Film for Label-free Electrochemical Bacterial Cell Detection

**DOI:** 10.1021/acssensors.2c01292

**Published:** 2022-09-02

**Authors:** Tina Skorjanc, Andraž Mavrič, Mads Nybo Sørensen, Gregor Mali, Changzhu Wu, Matjaz Valant

**Affiliations:** † Materials Research Laboratory, University of Nova Gorica, Vipavska 11c, 5270 Ajdovscina, Slovenia; ‡ Department of Physics, Chemistry and Pharmacy, University of Southern Denmark, Campusvej 55, 5230 Odense, Denmark; § NMR Center, National Institute of Chemistry, Hajdrihova 19, 1000 Ljubljana, Slovenia

**Keywords:** covalent organic polymers, electrophoresis, E. coli, electrochemical impedance spectroscopy, detection

## Abstract

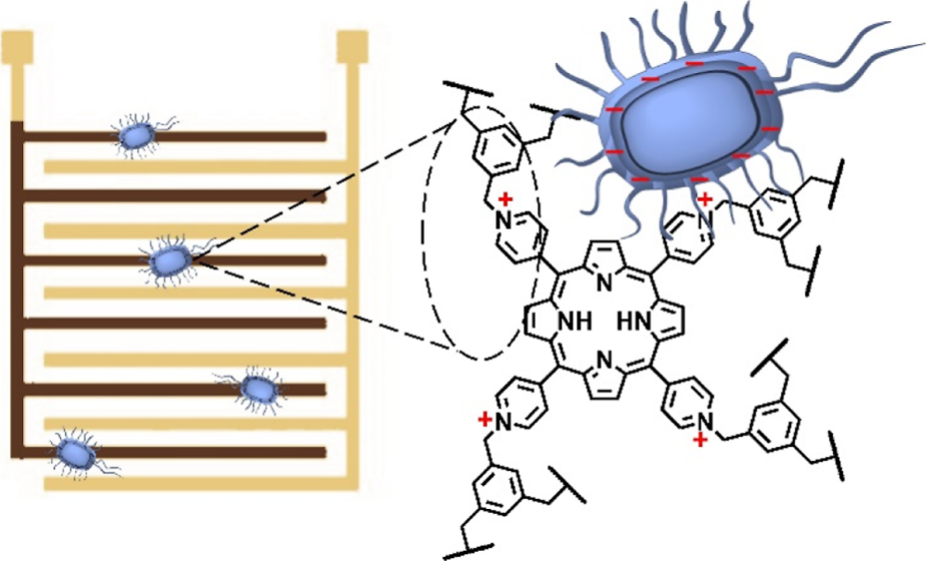

Numerous species of bacteria pose a serious threat to human health and cause several million deaths annually. It is therefore essential to have quick, efficient, and easily operable methods of bacterial cell detection. Herein, we synthesize a novel cationic covalent organic polymer (COP) named **CATN** through the Menshutkin reaction and evaluate its potential as an impedance sensor for *Escherichia coli* cells. On account of its positive surface charge (ζ-potential = +21.0 mV) and pyridinium moieties, **CATN** is expected to interact favorably with bacteria that possess a negatively charged cell surface through electrostatic interactions. The interdigitated electrode arrays were coated with **CATN** using a simple yet non-traditional method of electrophoresis and then used in two-electrode electrochemical impedance spectroscopy (EIS) measurements. The impedance response showed a linear relationship with the increasing concentration of *E. coli*. The system was sensitive to bacterial concentrations as low as ∼30 CFU mL^–1^, which is far below the concentration considered to cause illnesses. The calculated limit of detection was as low as 2 CFU mL^–1^. This work is a rare example of a COP used in this type of bacteria sensing and is anticipated to stimulate further interest in the synthesis of organic polymers for EIS-based sensors.

The primary infections of drug-resistant bacteria cause approximately five million deaths annually, making such infections the third leading cause of mortality globally.^1^ Moreover, secondary bacterial infections that follow various viral infections, such as Covid-19, are also lethal.^2^ Therefore, developing efficient bacteria detection methods is of great scientific, medical, forensic, biodefense, and food safety interest.^3^ Conventional methods of bacterial detection involve classical culturing techniques that require several handling steps or advanced scientific equipment, including polymerase chain reaction to detect nucleic acids and enzyme-linked immunosorbent assay to monitor antigen–antibody interactions.^4^ These methods are highly accurate and specific, but they also face several drawbacks. They are laborious, time-consuming, and expensive, require trained operators, and fail to detect microorganisms in real time or outside the laboratory.^5^ Thus, there is a pressing need for inexpensive, easily operable, rapid, label-free, and portable detection methods that give a quantitative readout.

Electrochemical impedance spectroscopy (EIS) is a powerful technique that meets the abovementioned requirements.^6^ EIS measurements are facile to perform, do not require labeling, exhibit compatibility with complex samples, and offer high reproducibility.^7^ For details on the working principle of EIS, the reader is directed to the literature.^8^ EIS sensors register changes in the electrical properties at their surface (capacitance or resistance) that are caused by the interaction between the analyte (e.g., bacterial cells) and the recognition element on the surface of the electrode.^9^ A crucial point in using EIS for bacterial detection is therefore the preparation of the sensor electrodes.

Recently, interdigitated electrode arrays (IDEAs) have gained interest on account of their enhanced sensitivity and miniaturization of sensor platforms.^10^ Materials containing recognition elements of interest for the EIS sensor applications are commonly deposited onto the electrodes by drop casting,^11^ spin coating,^12^ vapor deposition,^13^ or cyclic voltammetry (CV).^14^ These methods face several challenges: some result in unevenly deposited materials or demand high levels of sample stability, while others require the materials to be conductive. Herein, we present an alternative electrophoresis method^15^ for IDEA preparation in which a fine suspension of particles is subjected to an external electric field. The potential applied across the electrodes causes the particles from the suspension to deposit onto one of the electrodes, depending on their surface charge. This approach allows simple deposition on geometrically defined IDEAs without using a mask. Unlike conventional methods, it is suitable for non-conductive and insoluble materials. Given these strengths of electrophoresis, we used it to deposit a novel porphyrin-based cationic covalent organic polymer (COP) onto a commercially available IDEA.

Various materials have been tested as EIS sensors for bacterial cells, including inorganic nanoparticles,^16^ metal–organic frameworks (MOFs),^17^ molecularly imprinted polymers (MIPs),^18^ and block copolymers.^11^ These materials have achieved fairly low limits of detection (LODs), but each class faces its own challenges. MOFs often suffer from poor stability in aqueous media.^19^ The reliability of MIPs has been questioned due to nonspecific interactions with the nonimprinted surfaces.^20^ Antibodies used as components of some sensors are laborious to produce and purify, and they have limited stability.^21^ In contrast, insoluble COPs and covalent organic frameworks (COFs) have so far not been explored as EIS sensors for bacteria, yet they can overcome many of the challenges other classes of materials are facing.^22−24^ They are highly thermally and chemically stable, possess tunable structures, and have low toxicity due to purely organic structures. Thus, we herein synthesize and characterize a novel cationic COP based on porphyrin for bacterial cell sensing. We utilize the Menshutkin reaction between the pyridyl derivative of porphyrin and 1,3,5-tris(bromomethyl)benzene to gain a positively charged material that is anticipated to interact with the negatively charged bacterial surface. By means of electrophoresis, we deposit the material onto a simple commercially available IDEAs with spacing between the sensing and reference electrode in the range of 100 μm such that we obtain a coated sensing electrode and an uncoated reference electrode. Finally, this system is used as an EIS sensor for a model bacterium, *Escherichia coli* DH5α. An excellent level of sensitivity is observed with LOD calculated at 2 CFU mL^–1^. In comparison with other reported systems for EIS detection of *E. coli*, our material exhibits one of the lowest LODs, a property that speaks to its potential.

## Results and Discussion

**Figure 1:**
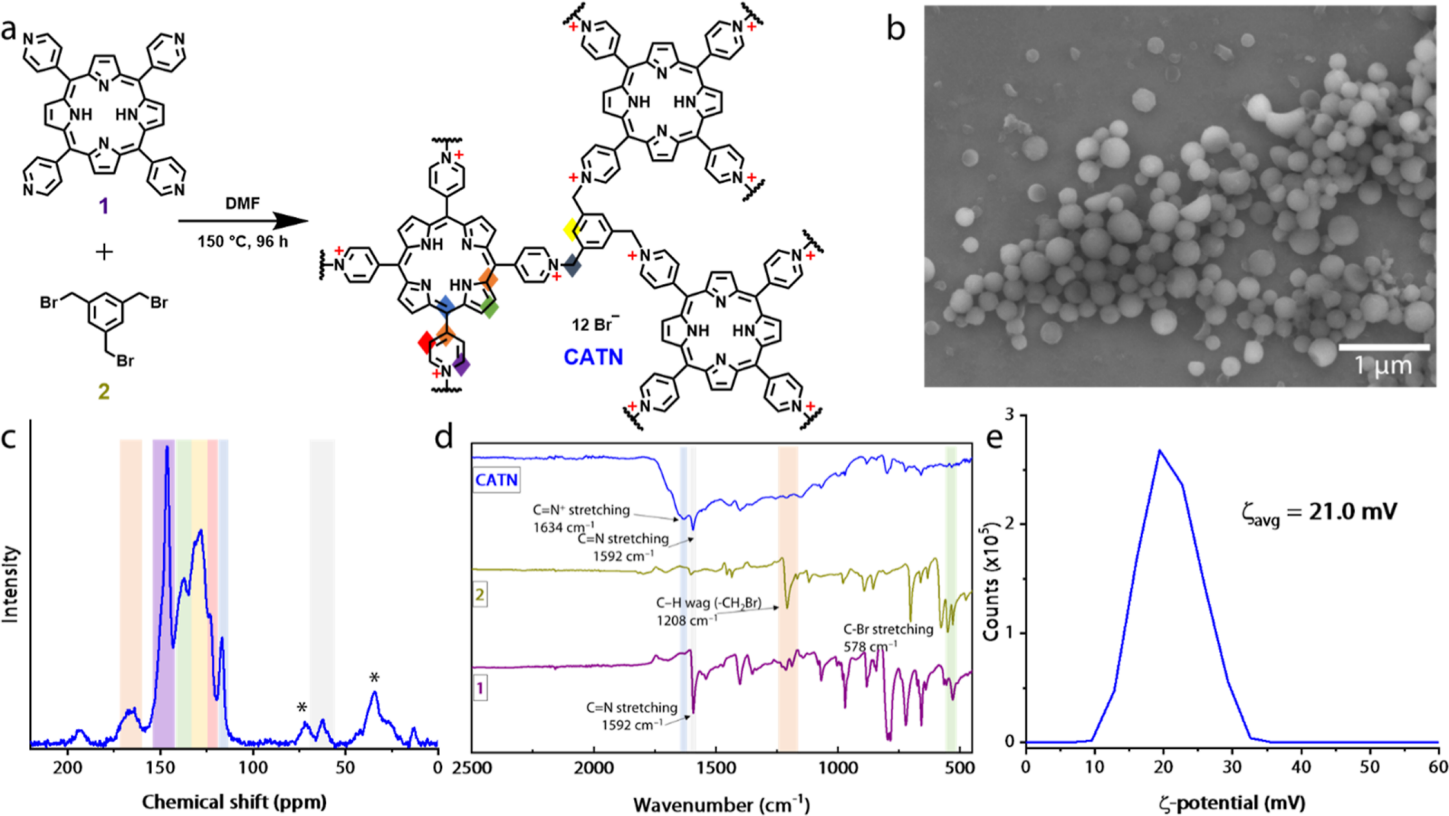
Design and characterization of **CATN**. (a) Synthetic scheme showing the preparation of **CATN**; (b) SEM micrograph of **CATN** showing spherical morphology; (c) cross-polarization magic-angle spinning (CP/MAS) solid-state ^13^C NMR spectrum of **CATN** with peaks assigned to C atoms in panel (a). Signals marked with * correspond to trapped solvents used in washing (DMF and CHCl_3_); (d) FT-IR spectra of **CATN** and its constituent building blocks; (e) ζ-potential measurements of **CATN** in water showing a positive surface charge.

The Menshutkin reaction is a common reaction in organic chemistry that uses a tertiary amine and an alkyl halide to generate a quaternary ammonium salt.^25^ In the current work, we utilized the pyridyl derivative of porphyrin [5,10,15,20-tetra(4-pyridyl)porphyrin] as a core and 1,3,5-tris(bromomethyl)benzene as a linker to generate a cationic network (**CATN**, Figure 1a). The reaction was carried out under reflux in anhydrous *N*,*N*′-dimethylformamide (DMF) for 96 h. The solids formed during the reaction were then purified by Soxhlet extraction using first DMF and then chloroform as extraction solvents. For achieving the best purity of **CATN**, it was soaked in CHCl_3_ overnight. After drying at 45 °C overnight, the **CATN** material was fully characterized.

Molecular-level characterization was performed with Fourier-transform infrared (FT-IR) spectroscopy and a cross-polarization magic-angle spinning (CP/MAS) solid state ^13^C NMR spectroscopy. The FT-IR spectra were recorded for both monomers and **CATN** (Figures 1d and S1 and S2). We note that the C–Br stretching and the C–H wagging from the CH_2_Br group are present in the linker at 578 and 1208 cm^–1^, respectively, but they do not appear in **CATN**, suggesting that the functional group has reacted. The C=N stretching appears both in the porphyrin derivative and in **CATN** at 1592 cm^–1^, as expected, due to the preservation of the porphyrin core structure. However, **CATN** exhibits an additional bond vibration at 1634 cm^–1^ that was ascribed to the C–N bonds within the quaternized pyridyl ring of the porphyrin. To further confirm the COP formation, we resorted to CP/MAS ^13^C NMR spectroscopy (Figure 1c). The recorded signals were ascribed to both building blocks of **CATN**. Several signals were assigned to the C atoms of the pyridyl rings, namely those at 165, 145, and 120 ppm. The porphyrin core shows a peak at 137 ppm that belongs to the pyrrole ring C atoms, and a peak at 117 ppm that was ascribed to the methine bridges. The C–N modes of the porphyrin showed a signal at ∼160 ppm, which is overlapping with one of the pyridyl ring signals (both marked by an orange diamond).^26^ Two of the signals correspond to the linker moiety, namely, a peak at 128 ppm assigned to the benzene ring and a peak at 63 ppm that corresponds to the aliphatic C atoms. Combined, the techniques of FT-IR and NMR spectroscopy confirmed the formation of **CATN** with a structure shown in Figure 1a.

Thermogravimetric analysis (TGA) was also performed with the building blocks and **CATN** (Figure S3). The measurements revealed that the polymerized network is thermally stable up to ∼300 °C, which is comparable to other covalent network structures.^27^ Powder X-ray diffraction (PXRD) measurements showed that the material is amorphous in nature (Figure S4). The amorphous nature of the material was further confirmed by a selected area electron diffraction measurement in a transmission electron microscope (TEM; Figure S5). Scanning electron microscopy (SEM) revealed a spherical shape of **CATN** particles (Figures 1b and S6). The particles had a diameter of ∼300 nm and formed larger clusters with a size of several microns. TEM imaging was performed to investigate the nature of these spheres (Figure S7). Images taken at different magnifications confirmed the spherical morphology and revealed that the **CATN** particles were solid spheres.

Based on the chemical reaction employed, it is anticipated that **CATN** would exhibit a positive surface charge. To confirm this, we performed ζ-potential measurements in water (Figure 1e). The overall net charge was found to be +21 mV, a property highly useful for the detection of bacteria. Bacterial cell surfaces possess a net negative electrostatic charge that originates from ionized phosphoryl and carboxylate substituents on the macromolecules in the outer cell envelope exposed to the extracellular environment.^28^ This means that **CATN** can form electrostatic interactions with the bacterial cells and is thus expected to be responsive to the presence of bacterial cells.

**Figure 2:**
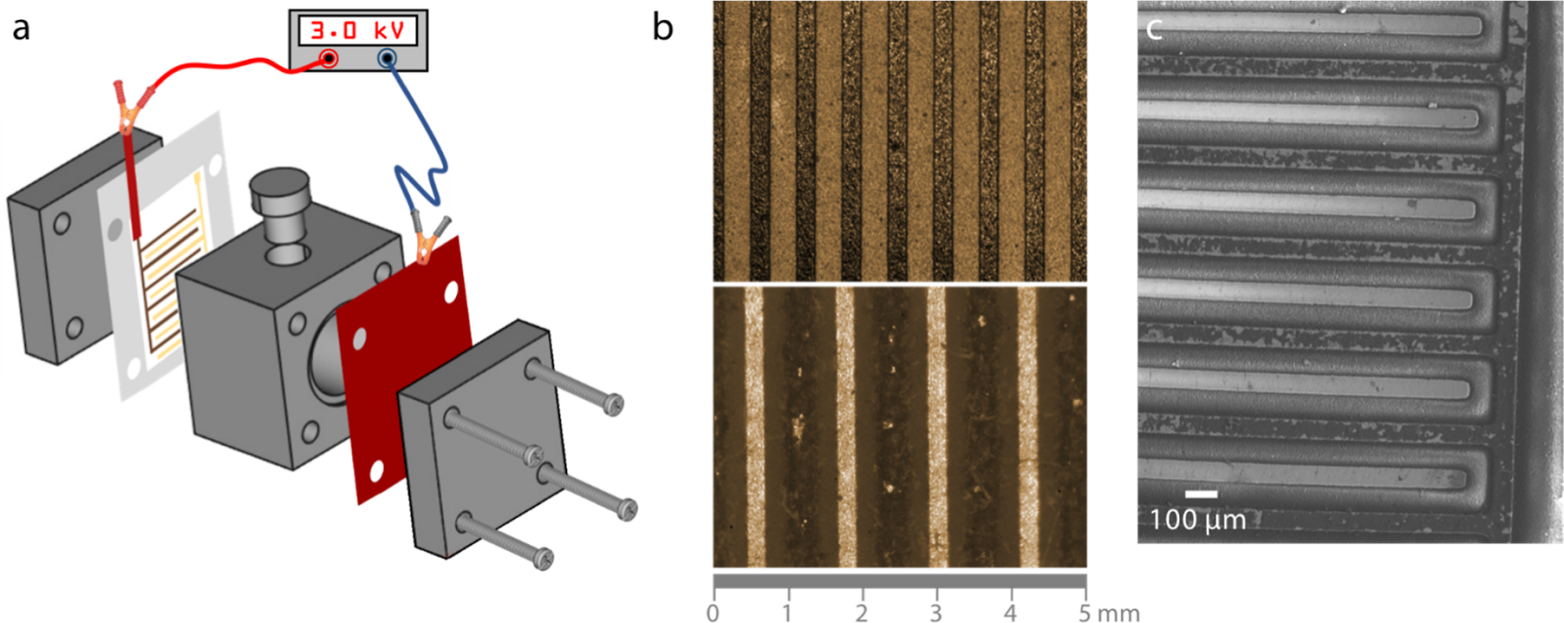
Preparation of the sensor electrode. (a) Schematic representation of the electrophoresis setup for **CATN** deposition onto a Au IDEA with Cu foil as a counter electrode; (b) optical microscopy images of the naked Au IDEA (top) and **CATN**-coated IDEA (bottom); (c) SEM micrograph showing a **CATN**-coated arm and a naked arm of the IDEA.

Having characterized the physical and chemical properties of **CATN**, we proceeded to depositing the material onto commercially available Au-coated IDEAs (Figure 2b bottom). To use the technique of electrophoresis, it was essential to prepare a stable suspension of **CATN**. An optimized set of conditions involved 30 min of sonication at 70% power in ethyl acetate (0.125 mg mL^–1^) followed by two rounds of centrifugation at 5000 rpm for 1 min. The thus-prepared suspension was stable and had a narrow particle size distribution in DLS measurements with a half-width of 20.1 nm (Figure S8). Based on 10 consecutive measurements, an average particle size was ∼300 nm ± 20 nm. Photographs of the suspensions demonstrate that the stability was enhanced with the centrifugation step, as the finest particles remained in the supernatant (Figure S9). The suspension was used in a custom-made Teflon electrophoretic cell with a volume of 9.5 mL (Figure 2a; details of the setup in the Supporting Information).

The coated-to-be IDEA served as one electrode, and a strip of Cu foil served as the opposite electrode. A potential difference of 3.0 kV was applied, and the electric field formed in the cell caused **CATN** particles to migrate from the suspension to the IDEA within 10 min. The electrophoretic cell was disassembled, and the IDEA was dried on air. Optical microscopy images of a bare IDEA and an IDEA with a **CATN**-coated arm are shown in Figure 2b. The deposition of dark brown particles is visible on one arm even by the naked eye. SEM micrographs further confirmed that the method resulted in an even coverage of one arm, while leaving the other arm of IDEA completely uncoated (Figure 2c). FT-IR spectroscopy was used to check for any changes in the chemical structure of **CATN** following the deposition (Figure S10). We note that all the major peaks corresponding to the network also appear in the coated IDEAs.

**Figure 3:**
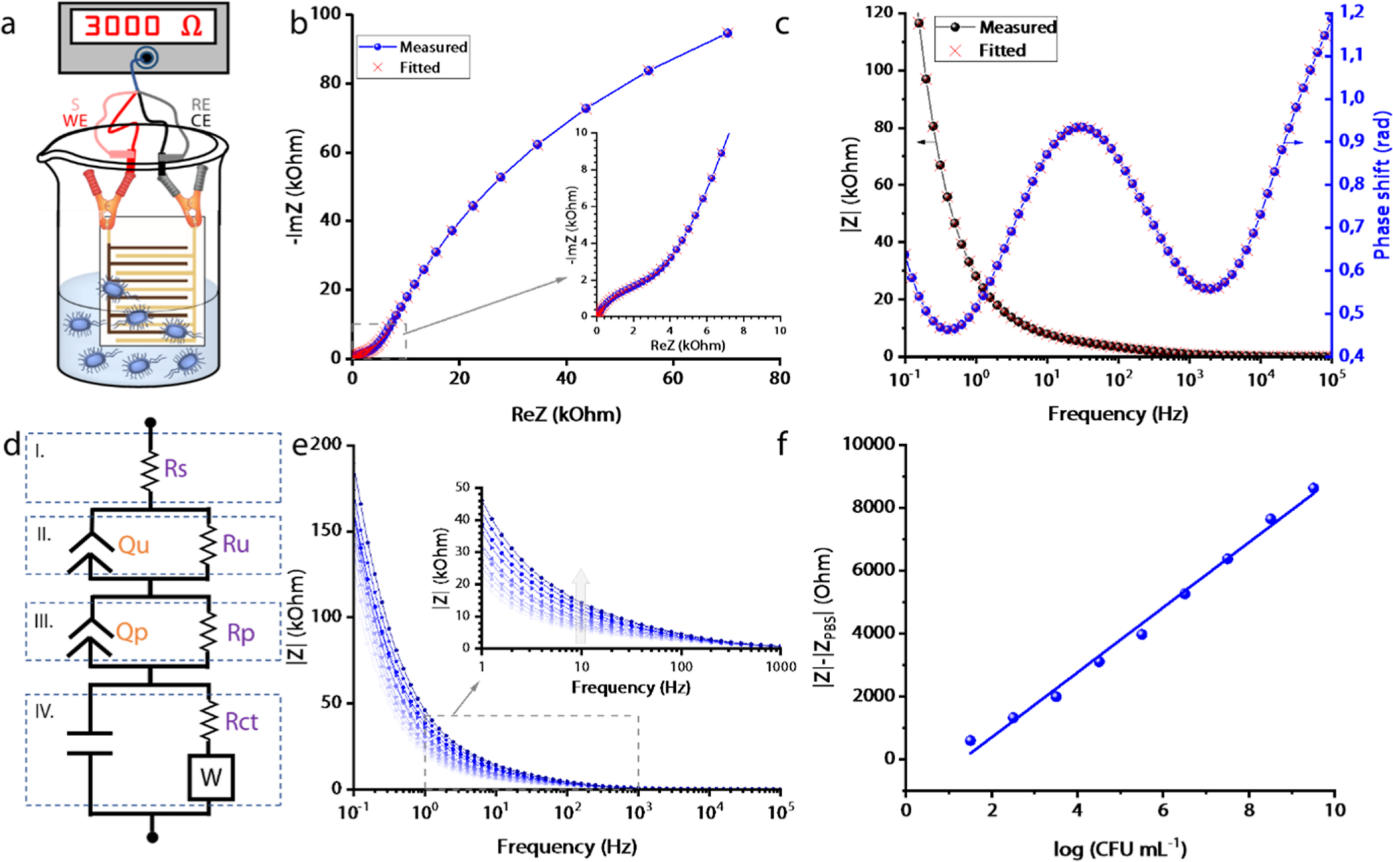
EIS detection of *E. coli* cells. (a) Schematic representation of the experimental setup. WE = working electrode, CE = counter electrode, S = sense, RE = reference electrode. (b) Nyquist plot showing experimental, and fitted real and imaginary components of impedance; (c) Bode plot showing experimental and fitted absolute impedance, and phase shifts as functions of frequency; (d) circuit diagram used in fitting the data shown in panels (b) and (c); (e) Bode plot showing a change in impedance as a function of frequency with increasing *E. coli* concentration; (f) linear relationship between the change of the impedance and the logarithm of the concentration of *E. coli* at 10 Hz. Line represents the linear regression curve: |*Z*| – |*Z*_PBS_| = 1033 log(CFU mL^–1^) – 1370; *R*^2^ = 0.992.

To evaluate the performance of the prepared **CATN**-coated IDEA in the detection of bacterial cells, *E. coli* (*E. coli*) strain DH5α was selected as a model organism. Since the negative surface charge is a general feature of any bacterial cell, we selected *E. coli* as a model on account of its wide availability.^29^ The bacterial colonies were cultured overnight until their optical density (O.D.) reached 1.0. The pellet was then collected and washed with phosphate buffered saline (PBS), and dilutions in the range of 10^–9^ to 10^–1^ were prepared (details in the Supporting Information). Figure 3a shows an experimental setup for the EIS measurements that were performed as a method of *E. coli* detection. The faradaic approach using Fe^2+/3+^ as a redox probe was selected over the non-faradaic one, as it is generally considered more sensitive.^30^ A two-electrode system was used in the potentiostatic EIS mode, and a potential of 150 mV was applied between the S and RE electrodes, corresponding to the Fe^2+^ oxidation potential (Figure S11a). Although the applied voltage can damage the bacterial cell membranes, this only happens at much higher voltages than those used in the current experiments.^31^ The EIS data for IDEA dipped in PBS are presented with the Nyquist and the Bode plots in Figure 3b,c. The EIS data were fitted using a model circuit shown in Figure 3d. The parameters obtained for the IDEA dipped into PBS are shown in Table S1 along with their relative errors; the residual plot of relative and imaginary part of the impedance is given in Figure S11b. The PBS solution was modeled as a resistor (Rs; labeled I), and its resistivity was found to be 44 Ω. The formation of the double layer on **CATN** is presented with a resistor and constant phase element in parallel (Rp|Qp; labeled III). The uncoated part of the electrode, which was directly accessible to electrolyte ions, was modeled as a resistor and constant phase element in parallel (Ru|Qu; labeled II). The resistivity of the **CATN** layer is almost 100-fold higher than that of the uncoated part at 232 kΩ versus 3 kΩ, respectively. The processes arising from the Fe^2+^^/^^3+^ redox probe at the electrode interface are presented with the Randles equivalent circuit as a capacitor in parallel with a series of a resistor for a charge transfer resistivity and a Warburg impedance element (C|(Rct-W); labeled IV). The charge transfer resistance for oxidation of Fe^2+^ to Fe^3+^ in the absence of bacteria was found to be 650 Ω, which agrees with previously reported values.^32,33^

EIS measurements in the presence of different concentration of *E. coli*. are presented in the Bode plot in Figure 3e. A general trend where an increase in the concentration results in an increase of the impedance signal in the range of 10 MHz to 100 Hz can be observed. This low-frequency range is associated with the charge transfer resistivity of Fe^2+^/^3+^ redox probe at the electrode/electrolyte interface and indicates that bacteria’s absorption to the electrode surface, as expected, suppresses the charge transfer. The change in the absolute impedance at 10 Hz shows a linear trend with respect to the logarithm of *E. coli* concentration expressed in CFU mL^–1^ (Figures 3f and S12). As can be noted in the graph, our system responded to concentrations of bacteria down to ∼30 CFU mL^–1^ and reached LOD of just 2 CFU mL^–1^. This value is well below what is considered an illness-causing concentration for various species of bacteria.^34^ It should be noted that only 5 min was required for each reading to stabilize which demonstrates the quick response time of our system.

Several examples of materials have been reported for EIS sensing of *E. coli* cells (Table S2). The majority of reported systems utilize bacterium-specific antibodies and are thus suitable for the sensing of a particular species of bacterium only. In contrast, **CATN** shows the ability to detect various bacterial species regardless of their Gram-positive or Gram-negative nature (Figures S13-S14). Most of the reported systems have higher limits of detection than **CATN**. Furthermore, antibodies are laborious to produce and purify, and they may have limited stability.^35,36^ Other systems with aptamers, carbohydrates, and lectins have also been reported with similarly higher limits of detection, but there is a severe lack of polymer systems explored for this type of application.

**Figure 4:**
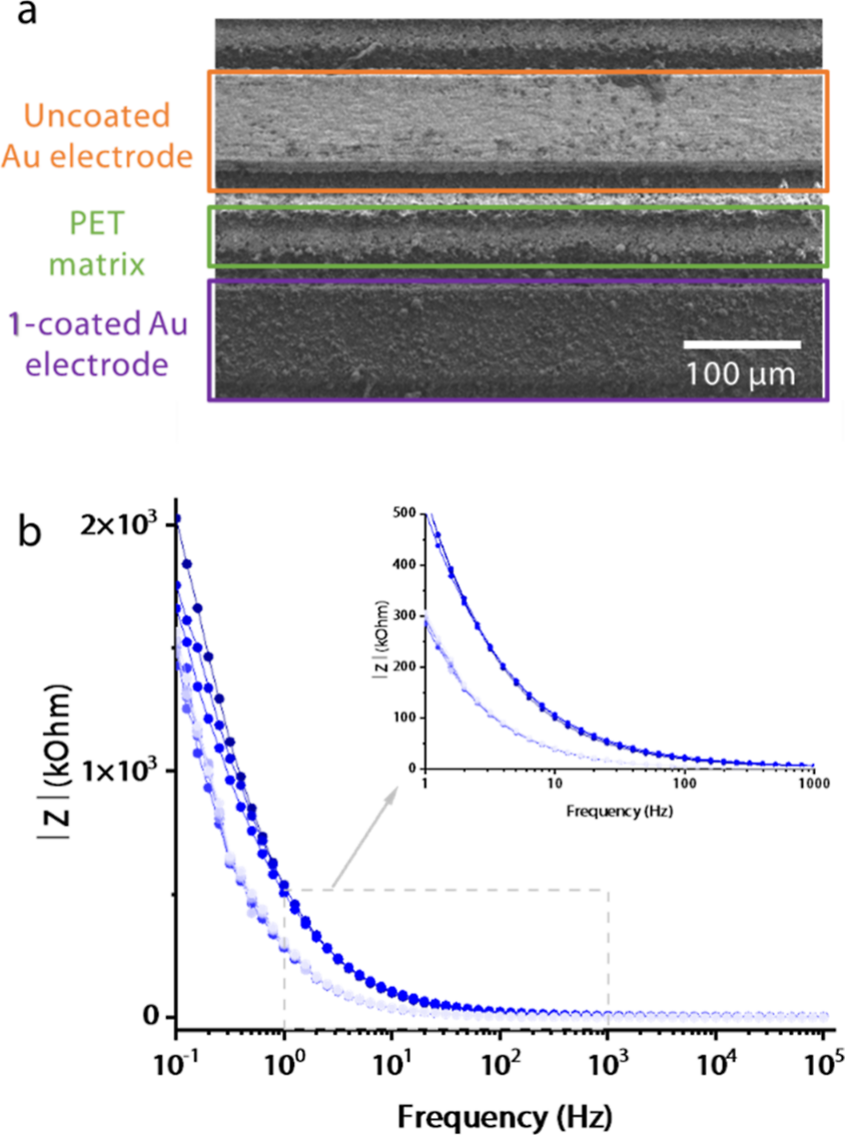
Experiment with the porphyrin monomer-coated IDEA. (a) SEM micrograph showing a zoomed-in section with an uncoated gold and a 5,10,15,20-tetra(4-pyridyl)porphyrin-coated electrodes of an IDEA; (b) Bode plot showing the impedance signal as a function of frequency with increasing *E. coli* concentration.

An experiment was also performed with the monomer **1** coated onto an IDEA in a similar fashion as **CATN** (details in the Supporting Information, Figure 4a). A sensing experiment with different concentrations of *E. coli* cells in PBS was carried out. In contrast to **CATN**, the porphyrin monomer **1** performed poorly (Figure 4b), as it was unable to show a response beyond the three initial runs. A possible explanation for this phenomenon is that the pyridyl functional groups with a free electron pair form an interaction with the Fe^2+^ ions of the redox mediator in the solution.^37^ Once the pyridyl sites are saturated, the response is no longer changing with an increasing *E. coli* concentration.

## Conclusions

In summary, this work introduced several novel concepts to the field of electrochemical sensing fabrication of bacteria. First, a new COP **CATN** was synthesized and fully characterized with molecular-level and macroscopic techniques. Second, electrophoresis as an alternative to the traditional methods of electrode preparation is demonstrated for an IDEA. It is found to be an efficient method that produces evenly coated surfaces even in miniaturized setups. Finally, EIS measurements with varying concentrations of *E. coli* bacterial cells showed a consistent response with a short stabilization time of 5 min and sensitivity for bacteria in very dilute solutions down to 30 CFU mL^–1^. It is anticipated that this study will stimulate further interest in the development of organic polymer-based electrochemical biosensors and in further exploring alternative methods of electrode preparation.

## Supporting Information Available

Synthetic procedures for the monomer and **CATN**, details of *E. coli* culturing, electrode preparation, thermogravimetric analysis, powder XRD, TEM images of **CATN**, DLS data, cyclic voltammetry data, EIS parameters, *E. coli* colony counting, performance of known EIS sensors for *E. coli* (PDF)

## Abbreviations

CFU: colony forming unit
COF: covalent organic framework
COP: covalent organic polymer
CP/MAS: cross-polarization magic-angle spinning
CV: cyclic voltammetry
DLS: dynamic light scattering
DMF: *N*,*N*′-dimethylformamide
EIS: electrochemical impedance spectroscopy
FT-IR: Fourier-transform infrared
IDEA: interdigitated electrode array
LOD: limit of detection
MIP: molecularly imprinted polymer
MOF: metal–organic framework
PXRD: powder X-ray diffraction
SEM: scanning electron microscopy
TEM: transmission electron microscopy
TGA: thermogravimetric analysis
