# Supplementary material for: Cationic Covalent Organic Polymer Thin Film for Label-free Electrochemical Bacterial Cell Detection
Source: ACS Sens. 2022 Sep 2;7(9):2743–9. doi: 10.1021/acssensors.2c01292 (PMC9513792; doi:10.1021/acssensors.2c01292)
Supplement: Supplementary file 1 — se2c01292_si_001.pdf [file se2c01292_si_001.pdf]

Supporting Information

# A cationic covalent organic polymer thin film for label-free electrochemical bacterial cell detection

Tina Skorjanc,[1] Andraž Mavrič,[1] Mads Nybo Sørensen,[2] Gregor Mali,[3] Changzhu Wu,*[2] and Matjaz Valant*[1]

[1] Materials Research Laboratory, University of Nova Gorica, Vipavska 11c, 5270 Ajdovscina, Slovenia

[2] Department of Physics, Chemistry and Pharmacy, University of Southern Denmark, Campusvej 55, 5230 Odense, Denmark

[3] NMR Center, National Institute of Chemistry, Hajdrihova 19, 1000 Ljubljana, Slovenia

Correspondence: wu@sdu.dk, matjaz.valant@ung.si

**General.** All chemicals and solvents were purchased from Sigma-Aldrich and used without further purification. Deionized water was used from Adrona B30 water purification system. The Au-coated IDEA – PET flexible fork electrodes (5 by 10 mm, 10 pairs of 100 µm line width line spacing) were purchased from Anni TKuke Store via the AliExpress platform. Routine nuclear magnetic resonance (NMR) spectra were recorded at 25 °C on a Bruker Avance spectrometer, with working frequencies of 400 MHz for $^1$H, and 100 MHz for $^{13}$C nuclei, respectively. All chemical shifts are reported in ppm relative to the signals corresponding to the residual non-deuterated solvent ($CDCl_3$: δ = 7.26 ppm).

**Materials Characterization.** Fourier-transform (FT-IR) spectra were recorded on the Perkin Elmer Spectrum 100 with an attenuated total reflectance (ATR) attachment. NMR measurements were carried out on a 600 MHz Varian NMR system equipped with a 1.6 mm Varian T3 HXY MAS probe. Larmor frequencies for $^1$H and $^{13}$C nuclei were 599.50 MHz, and 150.72 MHz, respectively. Sample rotation frequency was 20 kHz. $^1$H-$^{13}$C cross-polarization (CP) MAS NMR spectrum was recorded by first exciting protons and transferring polarization to carbon nuclei using the amplitude-ramped CP block with a duration of 4 ms. During the acquisition, high-power XiX heteronuclear decoupling was applied; repetition delay was 1 s and 53000 scans were collected. $^1$H-$^{13}$C CPMAS NMR spectrum was referenced to the corresponding signal of TMS. X-ray diffraction measurements were performed on Rigaku SmartLab II with Cu $K_\alpha$ (λ = 1.5405 Å) radiation source operating at 40 kV and 40 mA. The patterns were recorded with divergent slit of 1/16° over the 2θ range of 5–50° with step size = 0.03° and a scan speed of 0.3° min$^{-1}$. TGA experiments were performed on Mettler Toledo TGA/DSC2 with a heating rate of 10 °C min$^{-1}$ over a temperature range of 75–1000 °C. Elemental analysis was performed on Perkin Elmer Series II CHN 2400 analyser. Dynamic light scattering (DLS) experiments were performed on Brookhaven Instruments Corporation 90 Plus/BI-MAS using ethyl acetate as a solvent. ζ-potential measurements were carried out in Malvern Zetasizer NanoSeries using water as a solvent. Scanning electron microscopy (SEM) images were recorded on JEOL JSM-7001 TTLS operating at 4.0 kV. Powder samples were drop-cast on Si wafer substrates, and electrodes were imaged by attaching them directly to the sample holder. Transmission electron microscopy (TEM) images were collected on JEOL JEM-2100 HR operating at 200 kV. Optical microscopy imaging was performed on Nikon Eclipse microscope equipped with an Infinity camera.

**Synthesis of 5,10,15,20-tetra(4-pyridyl)porphyrin.** The synthesis was performed by following a published procedure with minor modifications.[1] 4-pyridinecarboxaldehyde (2.725 g, 25.4 mmol) was dissolved in propionic acid (150 mL) and heated to 140 °C. Pyrrole (1.714 g, 25.4 mmol) was added and the solution immediately started to darken. The mixture was stirred at 140 °C for 3 hours. The mixture was allowed to cool, and the solvent was removed *in vacuo*. The product was precipitated by addition of cold MeOH (100 mL) and left in the freezer overnight. The precipitate was filtered and rinsed with MeOH until the liquid became colorless. The product was isolated as a purple crystalline solid. Yield: 647 mg, 1 mmol, 16 %. $H^1$ NMR (400 MHz, $CDCl_3$) δ 9,06 (d, 8H), 8.87 (s, 8H), 8.17 (d, 8H), -2.92 (s, 2H).

**Synthesis of CATN.** The Menshutkin reaction was carried out between 5,10,15,20-tetra(4-pyridyl)porphyrin (30 mg, 0.050 mmol) and 1,3,5-tris(bromomethyl)benzene (23 mg, 0.065 mmol) using 3 mL anhydrous N,N-dimethylformamide (DMF) as a solvent. The reflux reaction was setup at 150 °C with continuous stirring and under $N_2$ atmosphere for four days. The precipitated solids were briefly washed and DMF and acetone, and thoroughly purified using Soxhlet extraction with DMF and chloroform used as solvents for 48 hours each. Finally, **CATN** was dried in an oven at 45 °C overnight as used for materials characterization. From a typical batch, an average of 17.9 mg ± 2.2 mg **CATN** was obtained. Elemental analysis: C: 59.2 %, H: 5.1 %, N: 12.0 %.

***E. coli* DH5α culturing.** A single colony was inoculated into 10 mL of sterilized Luria broth (LB) medium at 30 °C with shaking at 250 rpm overnight. When the optical density (O.D.) reached ~1.0, the bacterial cells were collected by centrifugation (5000 rpm, 10 min) and washed with 10 mL phosphate buffered saline (PBS) twice. Finally, the cells were resuspended in 10 mL PBS. For using them in sensing experiments, the cells were serially diluted to obtain suspensions with dilution factors ranging from $10^{-9}$ to $10^{-1}$.

To correlate the dilution factors to colony-forming units per mL (CFU $mL^{-1}$), 50 μL aliquots of each dilution were seeded onto separate agar plates and incubated at 37 °C overnight. The following day, the colonies were counted for the plates that had fewer than 200 colonies. Taking into account the dilution factors, the concentration of bacteria was expressed as CFU $mL^{-1}$.

***B. subtilis* culturing.** Similar to *E. coli*, *B. subtilis* cells were inoculated into 10 mL of sterile LB medium and cultured overnight under the same conditions. When the optical density (O.D.) reached ~1.0, the bacterial cells were collected by centrifugation (5000 rpm, 10 min) and washed with 10 mL phosphate buffered saline (PBS) twice. Finally, the cells were resuspended in 10 mL PBS. For using them in the EIS experiments, the cells were serially diluted to obtain suspensions with various dilution factors.

**CATN-coated electrode preparation and characterization.** Electrophoretic deposition was carried out using a custom-made Teflon electrophoretic cell and the PHYWE high voltage power supply. The cell was fabricated from Teflon to ensure compatibility with various solvents. Its inner volume was 9.5 mL, and it consisted of three major parts as indicated in Figure 2: the central body where the suspension was pipetted (3x4x4 cm) and two side panels to enclose the liquid (1x4x4 cm each). The three parts were connected by four screws (#8, 6 cm in length) and tightened with the corresponding nuts. To prevent leaking of the suspension, an O-ring made of Viton® rubber was used between the central body and each of the side panels.

The suspensions for electrophoresis were prepared using the Bandelin Sonoplus ultrasonicator with the UW-100 tip operating at 70 % power. Cyclic voltammetry and electrochemical impedance spectroscopy were done using ZIVE SP1 Potentiostat/galvanostat/EIS instrument (WonATech, Seoul, Korea). Electrochemical measurements were done in two electrode setups

with interdigitated electrode array (IDEA) sensor immersed into PBS electrolyte. Potassium hexacyanoferrate ($K_4Fe(CN)_6$, 5 mM) was used as a redox probe. EIS response was measured in the potentiostatic mode at +150 mV and an amplitude 10 mV in a frequency range of 100 kHz to 100 mHz. Uncoated Au electrode was used as a quasi-reference electrode and **CATN**-coated electrode was used as a working electrode. Impedance spectra was fitted with EIS Spectrum Analyser 1.0 using Powell minimization algorithm with parametric function to minimize.[2]

**Monomer-coated electrode preparation.** To prepare a 5,10,15,20-tetra(4-pyridyl)porphyrin-coated IDEA, 2.5 mg of the monomer was dispersed in 20 mL of ethyl acetate with the same sonication and centrifugation parameters as for **CATN**. Electrophoretic deposition was then carried out in the same Teflon-made cell at 2.0 kV, and complete coverage was achieved within 6 minutes. The electrode was inspected under both optical and electron microscopes before being utilized in a sensing experiment.

**Limit of detection (LOD)** was calculated using the formula

$$LOD = 3.3 \cdot \sigma / S,$$

where σ is the standard deviation of the electrochemical response signal

and S is the slope of the line-of-best-fit.

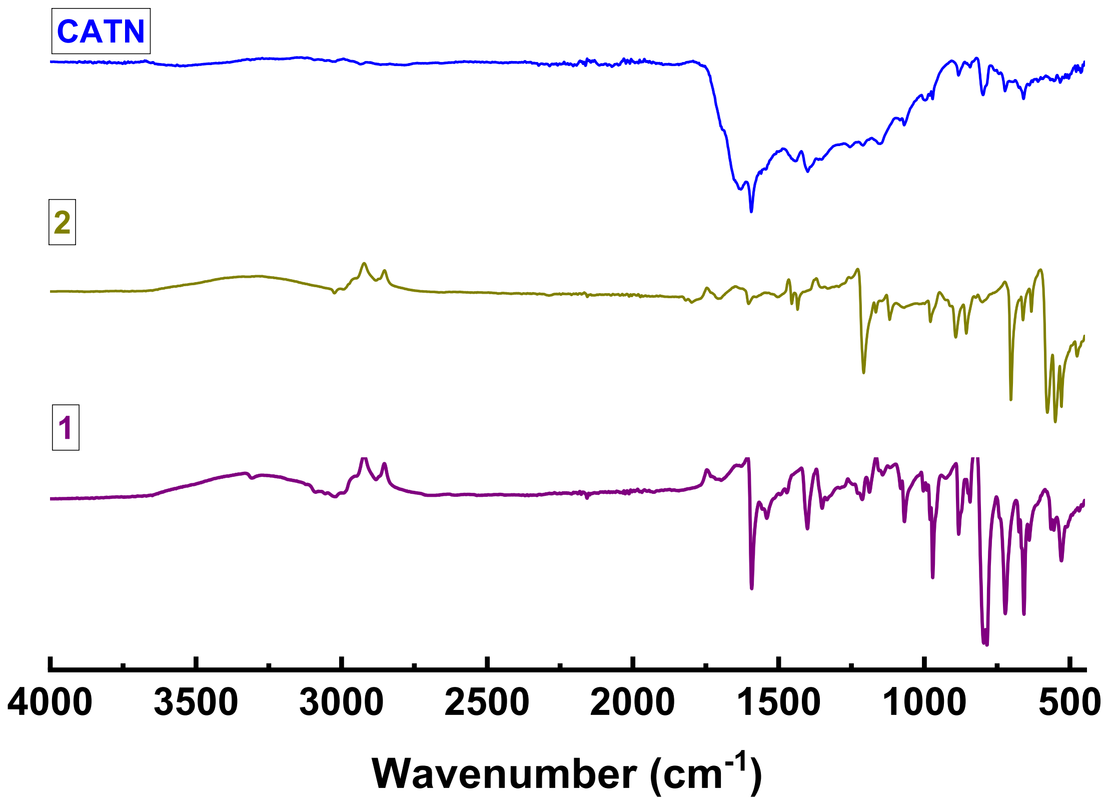


**Figure S1.** FT-IR spectra of **CATN** and its constituent building blocks in the 400 to 4000 $cm^{-1}$ range.

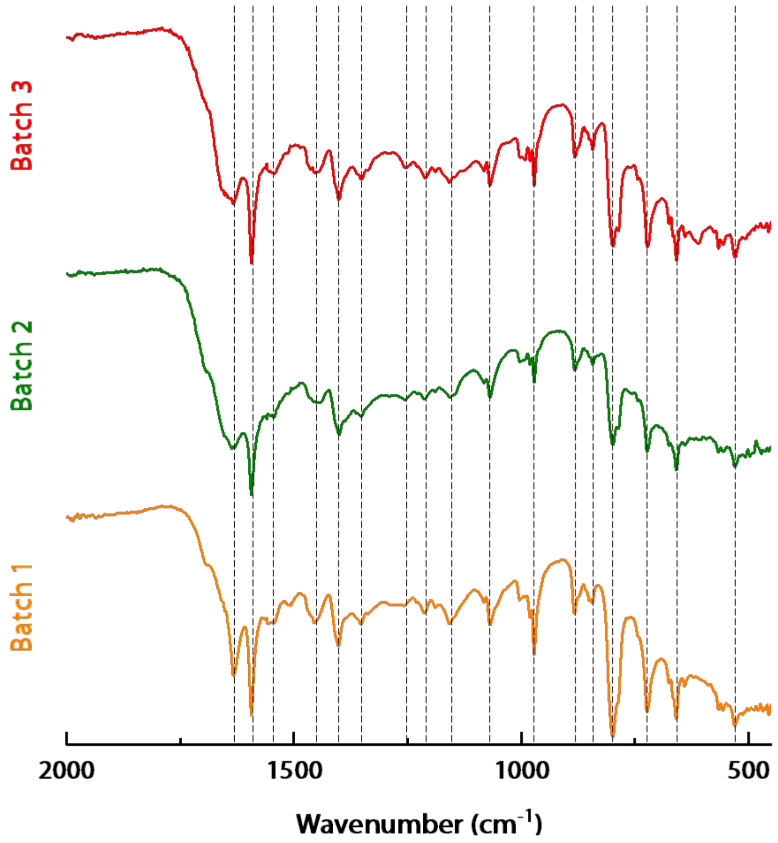


**Figure S2.** FT-IR spectra of **CATN** in three different batches of synthesis.

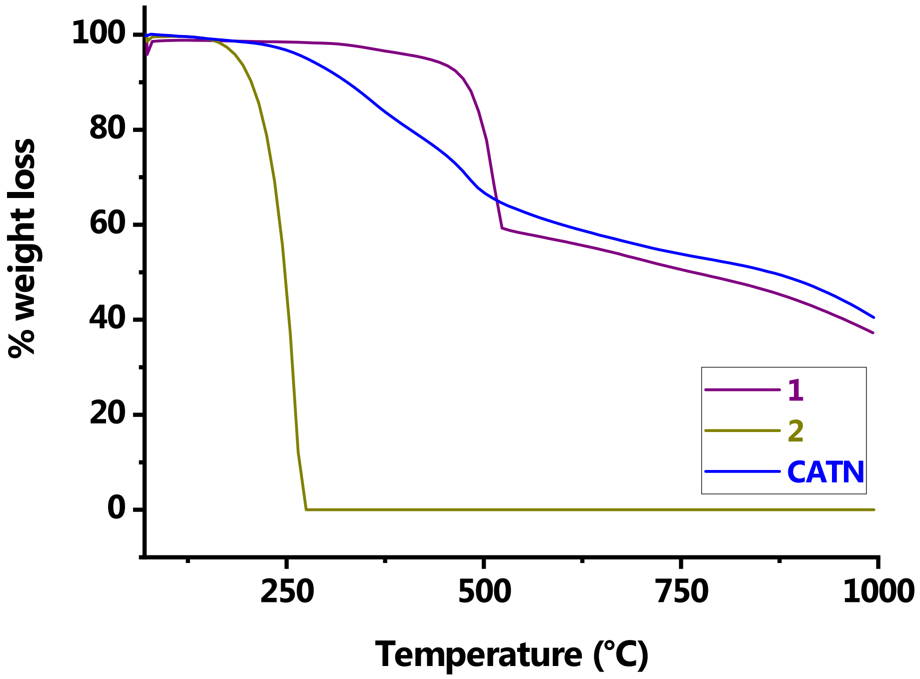


**Figure S3.** TGA profiles of the building blocks and **CATN**. All samples were first equilibrated at 75 °C to remove any trapped solvent molecules, and the measurements were run from 75 °C to 1000 °C.

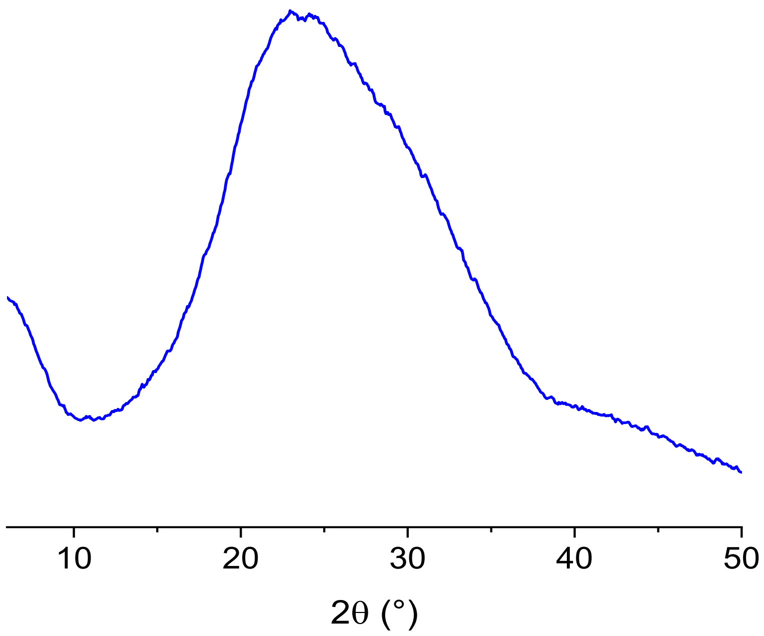


**Figure S4.** Powder X-ray diffraction pattern of **CATN**.

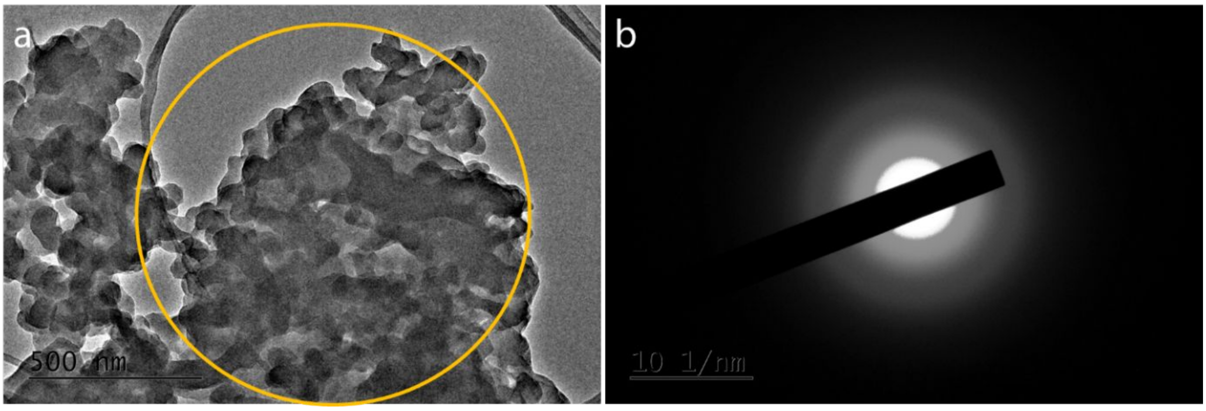


**Figure S5.** A TEM image of purified **CATN** (a) and SAED measurement (b) corresponding to the area marked by a yellow circle in panel (a).

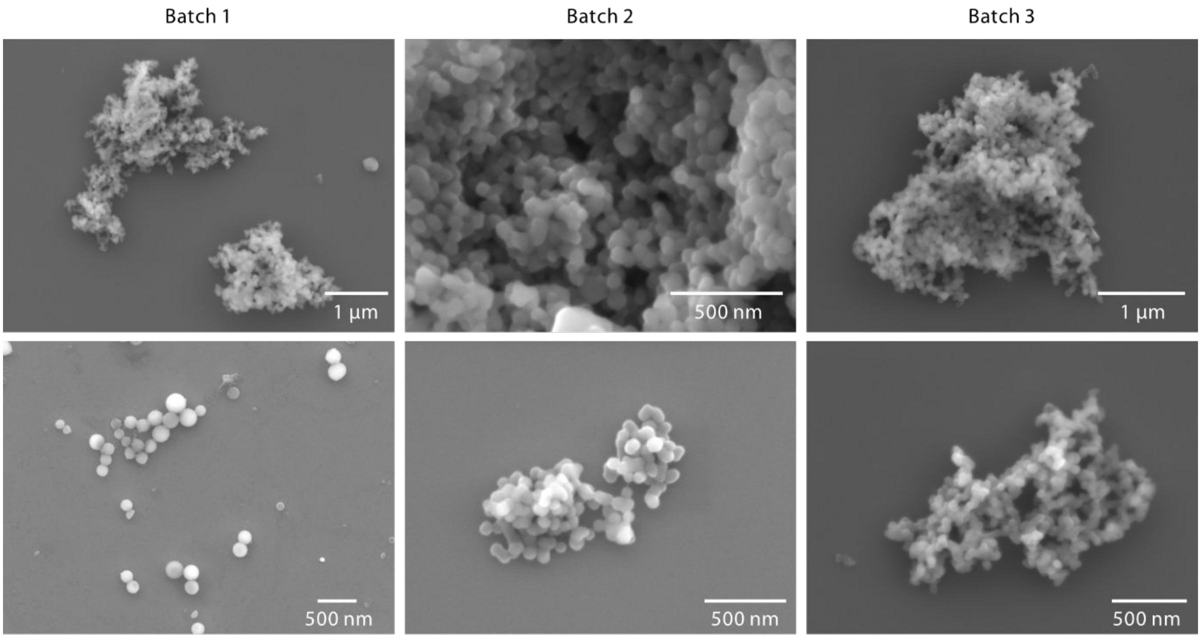


**Figure S6.** SEM micrographs of **CATN** obtained in various batches of synthesis.

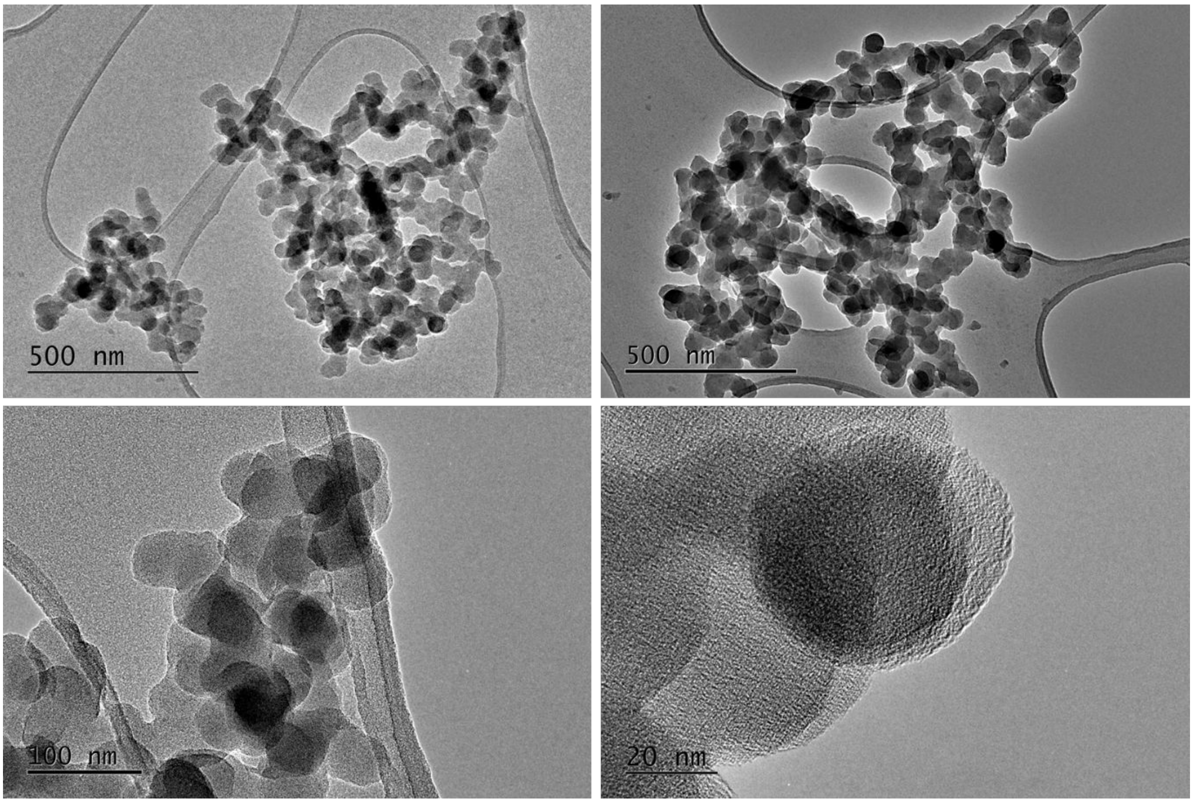

**Figure S7.** TEM micrographs of **CATN** on a holey carbon grid at different magnifications.

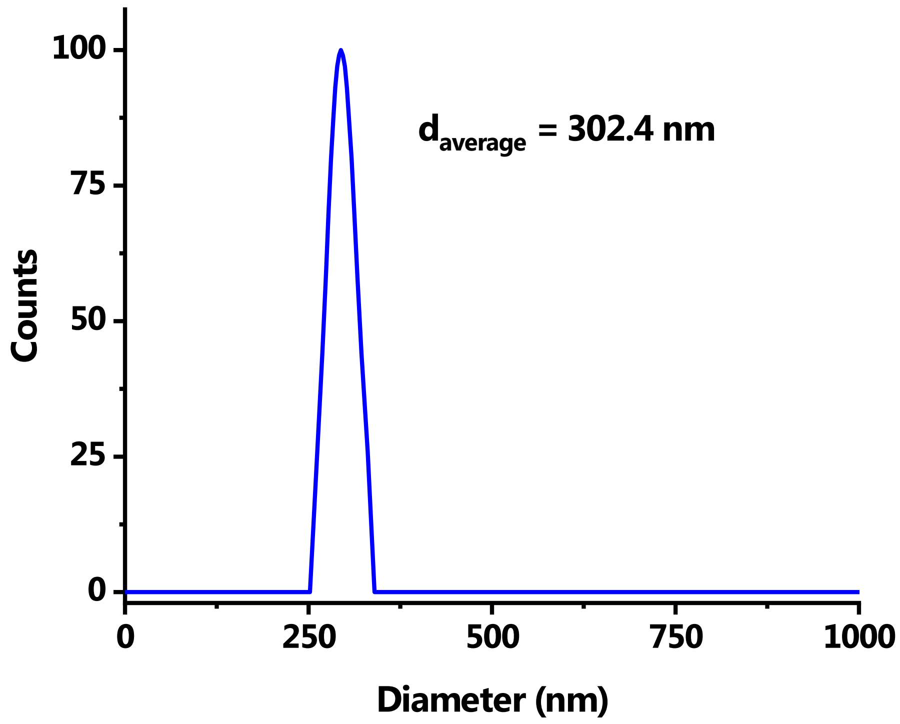


**Figure S8.** DLS particle size distribution in **CATN** suspension used for electrophoresis.

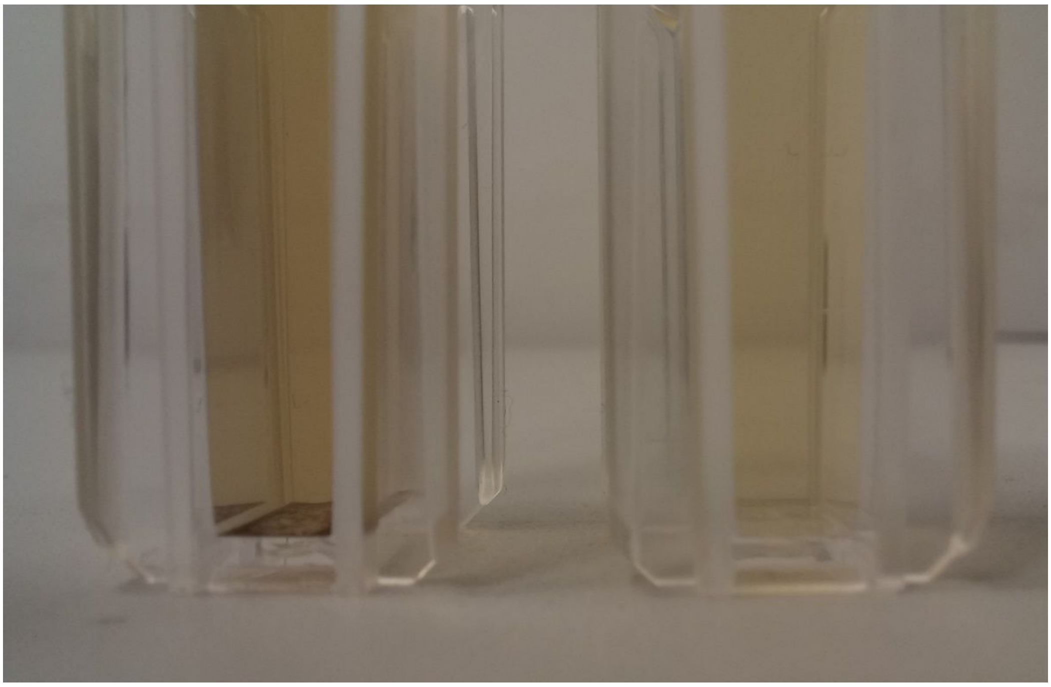

**Figure S9.** An optical photograph showing the **CATN** suspension after sonication (left) and after sonication + centrifugation treatment (right) several hours after suspension preparation.

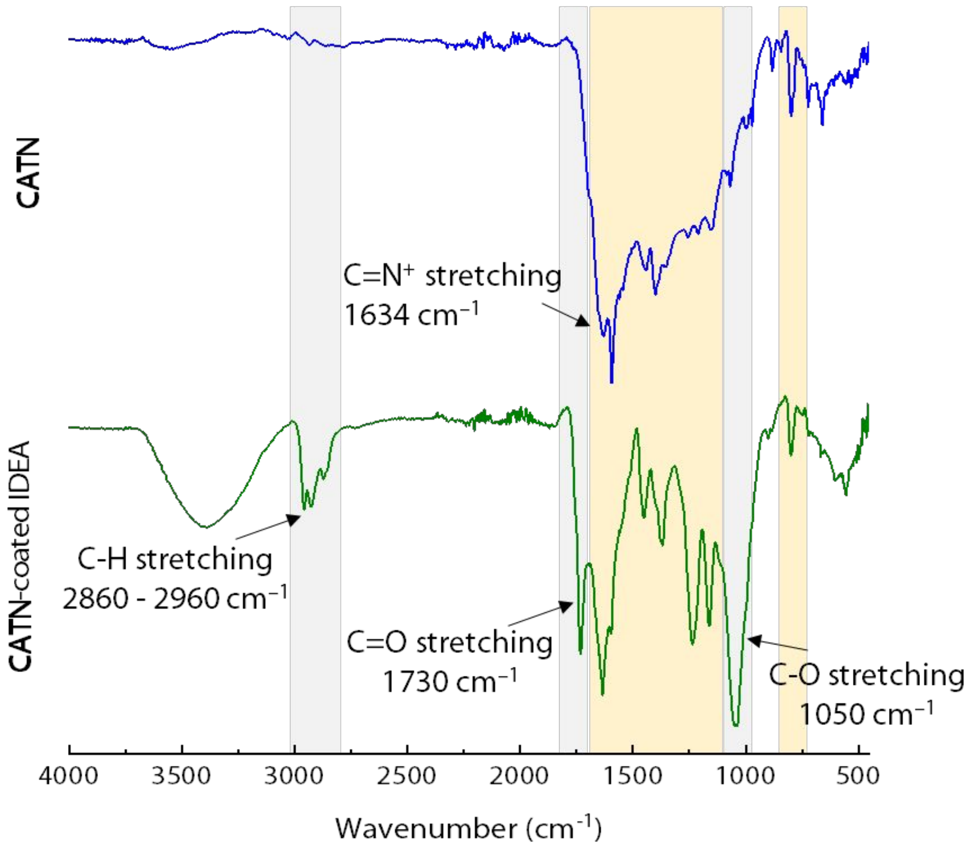


**Figure S10.** FT-IR spectra of **CATN,** and an IDEA coated with **CATN**. The yellow areas indicate signals corresponding to **CATN** in both samples, while the light gray areas correspond to the PET matrix of the IDEA.[3]

## Electrochemical measurements

**Table S1.** Fitted parameters and the corresponding relative errors for sensor electrode in PBS solution using equivalent circuit in Figure 3d. Measured and fitted date presented in Figure 3b-c. The residual plot of fitted data is presented in Figure S11.

| Element | Value | Error [%] |
|---|---|---|
| Rs | 0.044 kOhm | 2.7 |
| Pu | 8.95 uF | 1.4 |
| nu | 0.641 | 0.2 |
| Ru | 3.163 kOhm | 3.0 |
| Pp | 12.9 uF | 1.5 |
| np | 0.91 | 0.5 |
| Rp | 232.2 kOhm | 6.7 |
| Cdl | 0.58 uF | 3.6 |
| Rct | 0.65 kOhm | 5.9 |
| W | 8951 | 3.3 |

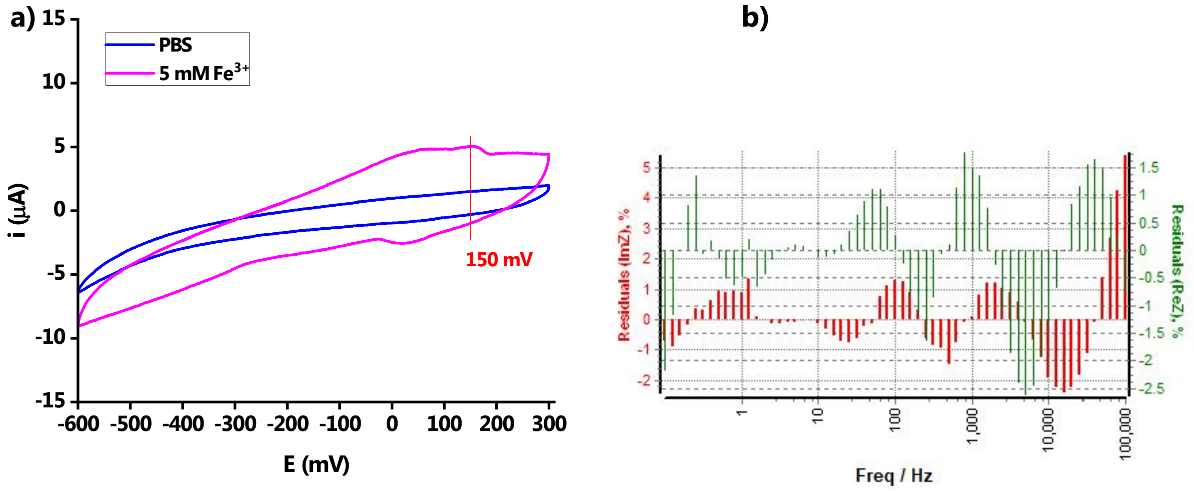


**Figure S11.** a) Cyclic voltammetry (CV) measurements in PBS (blue) and in 5 mM $Fe^{3+}$ (pink); b) residual plot for imaginary and real part of the impedance for fitted data in Figure 3 of the main text.

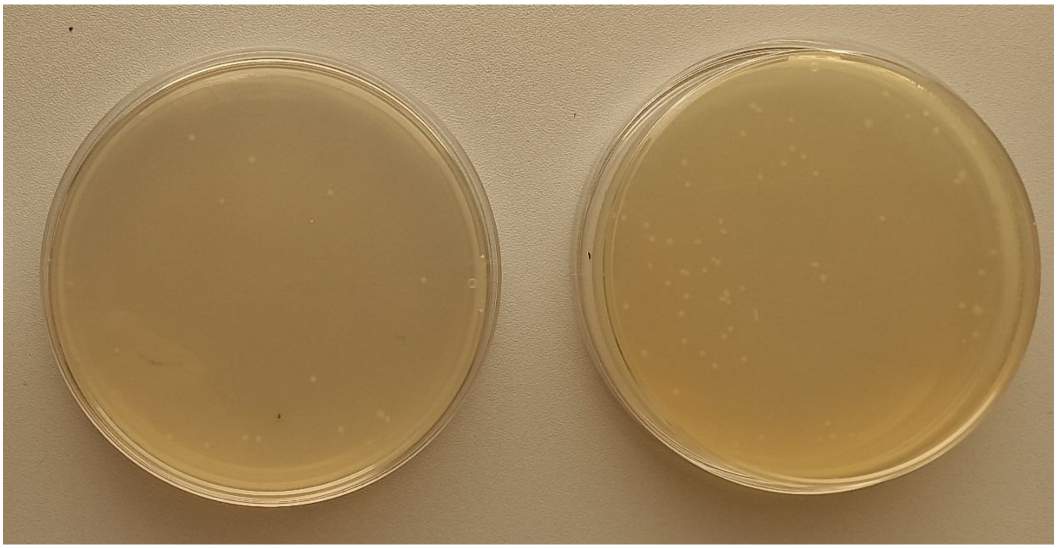

**Figure S12.** Examples of agar plates on which *E. coli* colonies were grown overnight to determine their concentrations in CFU $mL^{-1}$.

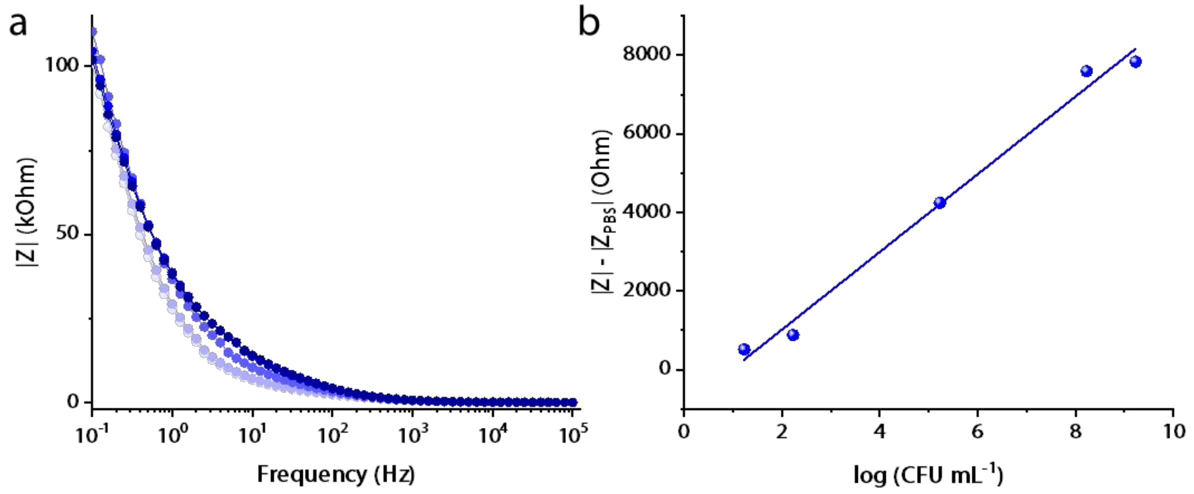


**Figure S13.** Results of a device-to-device reproducibility test. a) Bode plot showing a change in impedance as a function of frequency with increasing *E. coli* concentration; b) linear relationship between change of the impedance and the logarithm of concentration of *E. coli* at 10 Hz. Line represents linear regression curve: $|Z|-|Z_{PBS}|$ = 988,08 log(CFU $mL^{-1}$) – 955.66; LOD = 3, $R^2$ = 0.99.

**Table S2.** The performance of known *E. coli* sensors based on EIS.

| **Material** | **Limit of detection (CFU $mL^{-1}$)** | **Ref.** |
|---|---|---|
| *E. coli* antibodies immobilized on Au surface | 30 | [4] |
| *E. coli* antibodies immobilized on Au surface | 2 | [5] |
| *E. coli* antibodies on a dendrimer-covered electrode | 3 | [6] |
| *E. coli* antibodies immobilized on ITO surface | 6000 cells /mL | [7] |
| *E. coli* antibodies immobilized on ITO surface | $10^6$ | [8] |
| Self-assembled Au nanoparticles grafted with antibodies | 6000 cells /mL | [9] |
| Carbazide-PEG-thiol monolayer on Au surface | 12 | [10] |
| 3D Ag nanoflowers | 100 | [11] |
| Aptamer on $TaSi_2$ electrodes (3D IDEA) | 100 | [12] |
| Graphene-silk-antimicrobial peptides | 1000 | [13] |
| Lectin functionalized mixed self-assembled monolayer on gold electrode | 75 | [14] |
| Glycosylated quinone-fused polythiophene | 1700 | [15] |
| 3D printed biosensor | 53 | [16] |
| Metal-organic framework-polyalanine composite | 2 | [17] |
| P3HT-*b*-P3TEGT block copolymer | 500 | [18] |
| **CATN** | 2 | This work |

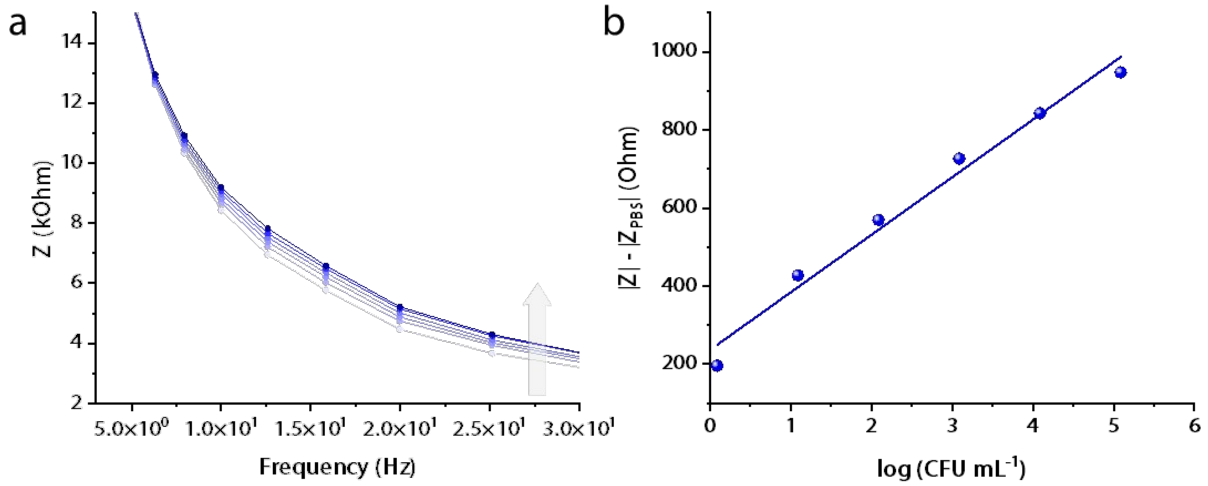


**Figure S14.** EIS experiments with *B. subtilis* as a model organism. a) Bode plot showing a change in impedance as a function of frequency with increasing *B. subtilis* concentration; b) linear relationship between change of the impedance and the logarithm of concentration of *B. subtilis* at 10 Hz. Line represents linear regression curve: $|Z|-|Z_{PBS}| = 147 \log(\text{CFU mL}^{-1}) + 237$; $R^2 = 0.98$.

## References


(1) Liu, S.; Tian, R.; Xu, J.; Wang, L.; Sun, J.; Jiang, X.; Wang, T.; Li, X.; Luo, Q.; Liu, J. Cucurbit[8]Uril-Based Supramolecular Nanocapsules with a Multienzyme-Cascade Antioxidative Effect. *Chem. Commun.* **2019**, *55* (92), 13820–13823.

(2) Bondarenko, A. S.; Ragoisha, G. A. Inverse Problem In Potentiodynamic Electrochemical Impedance. In *Progress in Chemometrics Research*; 2005; pp 89–102.

(3) Pereira, A. P. dos S.; Silva, M. H. P. da; Lima, É. P.; Paula, A. dos S.; Tommasini, F. J. Processing and Characterization of PET Composites Reinforced with Geopolymer Concrete Waste. *Mater. Res.* **2017**, *20*, 411–420.

(4) Cimafonte, M.; Fulgione, A.; Gaglione, R.; Papaianni, M.; Capparelli, R.; Arciello, A.; Bolletti Censi, S.; Borriello, G.; Velotta, R.; Della Ventura, B. Screen Printed Based Impedimetric Immunosensor for Rapid Detection of Escherichia Coli in Drinking Water. *Sensors*. 2020.

(5) Barreiros dos Santos, M.; Agusil, J. P.; Prieto-Simón, B.; Sporer, C.; Teixeira, V.; Samitier, J. Highly Sensitive Detection of Pathogen Escherichia Coli O157:H7 by Electrochemical Impedance Spectroscopy. *Biosens. Bioelectron.* **2013**, *45*, 174–180.

(6) Malvano, F.; Pilloton, R.; Albanese, D. Sensitive Detection of Escherichia Coli O157:H7 in Food Products by Impedimetric Immunosensors. *Sensors (Basel).* **2018**, *18* (7), 2168.

(7) Ruan, C.; Yang, L.; Li, Y. Immunobiosensor Chips for Detection of Escherichia c Oli O157: H7 Using Electrochemical Impedance Spectroscopy. *Anal. Chem.* **2002**, *74* (18), 4814–4820.

(8) Yang, L.; Li, Y.; Erf, G. F. Interdigitated Array Microelectrode-Based Electrochemical Impedance Immunosensor for Detection of Escherichia Coli O157:H7. *Anal. Chem.* **2004**, *76* (4), 1107–1113.

(9) Wan, J.; Ai, J.; Zhang, Y.; Geng, X.; Gao, Q.; Cheng, Z. Signal-off Impedimetric Immunosensor for the Detection of Escherichia Coli O157:H7. *Sci. Rep.* **2016**, *6* (1), 19806.

(10) Klass, S. H.; Sofen, L. E.; Hallberg, Z. F.; Fiala, T. A.; Ramsey, A. V; Dolan, N. S.; Francis, M. B.; Furst, A. L. Covalent Capture and Electrochemical Quantification of Pathogenic E. Coli. *Chem. Commun.* **2021**, *57* (20), 2507–2510.

(11) Huang, H.; Liu, M.; Wang, X.; Zhang, W.; Yang, D.-P.; Cui, L.; Wang, X. Label-Free 3D Ag Nanoflower-Based Electrochemical Immunosensor for the Detection of Escherichia Coli O157: H7 Pathogens. *Nanoscale Res. Lett.* **2016**, *11* (1), 1–8.

(12) Brosel-Oliu, S.; Ferreira, R.; Uria, N.; Abramova, N.; Gargallo, R.; Muñoz-Pascual, F.-X.; Bratov, A. Novel Impedimetric Aptasensor for Label-Free Detection of Escherichia Coli O157:H7. *Sensors Actuators B Chem.* **2018**, *255*, 2988–2995.

(13) Mannoor, M. S.; Tao, H.; Clayton, J. D.; Sengupta, A.; Kaplan, D. L.; Naik, R. R.; Verma, N.; Omenetto, F. G.; McAlpine, M. C. Graphene-Based Wireless Bacteria Detection on Tooth Enamel. *Nat. Commun.* **2012**, *3* (1), 763.

(14) Yang, H.; Zhou, H.; Hao, H.; Gong, Q.; Nie, K. Detection of Escherichia Coli with a Label-Free Impedimetric Biosensor Based on Lectin Functionalized Mixed Self-Assembled Monolayer. *Sensors Actuators B Chem.* **2016**, *229*, 297–304.

(15) Ma, F.; Rehman, A.; Liu, H.; Zhang, J.; Zhu, S.; Zeng, X. Glycosylation of Quinone-Fused Polythiophene for Reagentless and Label-Free Detection of E. Coli. *Anal. Chem.* **2015**, *87* (3), 1560–1568.

(16) Malhotra, S.; Pham, D. S.; Lau, M. P. H.; Nguyen, A. H.; Cao, H. A Low-Cost, 3D-Printed Biosensor for Rapid Detection of Escherichia Coli. *Sensors* **2022**, *22* (6), 2382.

(17) Gupta, A.; Bhardwaj, S. K.; Sharma, A. L.; Kim, K.-H.; Deep, A. Development of an Advanced Electrochemical Biosensing Platform for E. Coli Using Hybrid Metal-Organic Framework/Polyaniline Composite. *Environ. Res.* **2019**, *171*, 395–402.

(18) Elgiddawy, N.; Ren, S.; Yassar, A.; Louis-Joseph, A.; Sauriat-Dorizon, H.; El Rouby, W. M. A.; El-Gendy, A. O.; Farghali, A. A.; Korri-Youssoufi, H. Dispersible Conjugated Polymer Nanoparticles as Biointerface Materials for Label-Free Bacteria Detection. *ACS Appl. Mater. Interfaces* **2020**, *12* (36), 39979–39990.
